# Knowledge, attitude and practice survey of COVID-19 pandemic in Northern Nigeria

**DOI:** 10.1371/journal.pone.0245176

**Published:** 2021-01-14

**Authors:** Maryam Abdulrazaq Habib, Farouq Muhammad Dayyab, Garba Iliyasu, Abdulrazaq G. Habib

**Affiliations:** 1 Department of Internal Medicine, Murtala Mohammed Specialist Hospital, Kano, Kano State, Nigeria; 2 Infectious Disease Hospital, Kano, Kano State, Nigeria; 3 Infectious and Tropical Diseases Unit, College of Health Sciences, Bayero University, Kano, Kano State, Nigeria; Jouf University, Kingdom of Saudi Arabia, SAUDI ARABIA

## Abstract

**Background:**

A pandemic of coronavirus disease 2019 (COVID-19) emerged and affected most of the world in early 2020. To inform effective public health measures we conducted a knowledge, attitude and practice (KAP) survey among a Hausa Muslim society in Nigeria in March 2020.

**Methods:**

The study is an analytic cross-sectional survey with questionnaires administered to the general population including Health Care Workers (HCW) in Kano, Nigeria. Participants were recruited by convenience sampling following informed consent. The percentage of KAP scores were categorized as good and poor. Independent predictors of good knowledge of COVID 19 were ascertained using a binary logistic regression model.

**Results:**

The questionnaire was administered among urban 32.8%, peri-urban dwellers 32.4%, and to online participants 34.8%. The peri-urban and urban participants were given paper questionnaires. There were 886 study participants with mean age 28.58yrs [SD:10.25] (Interquartile range [IQR]:22yrs–32yrs), males 55.4% with 57.3% having had or were in tertiary education. Most participants were students 40% and civil servants 20%. The overall mean [standard deviation (SD)] for knowledge, attitude and practice scores expressed in percentage was 65.38%[SD15.90], 71.45% [SD14.10], and 65.04% [SD17.02] respectively. Out of the respondents, 270(30.47%) had good knowledge (GK), 158(17.8%) had good attitude (GA), and 230(25.96%) had good practice (GP) using cut-off scores of 75%, 86.5%, and 75% respectively. Over 48% did not agree COVID-19 originated from animals while 60% perceived the pandemic to be due to God’s punishment. Also, 36% thought it was a man-made virus. When rating fear, most respondents [63.5%] had marked fear i.e. ≥ 7 out of 10 and 56% admitted to modifying their habits recently in fear of contracting the virus. As regards attitude to religious norms, 77.77% agreed on cancellation of the lesser pilgrimage as a measure to curb the spread of the disease while 23.64% admitted that greater pilgrimage (Hajj) should proceed despite the persistence of the ongoing pandemic. About 50% of the respondents insisted on attending Friday congregational prayers despite social distancing. One in four people still harbored stigma towards a person who has recovered from the virus. 28% felt some races are more at risk of the disease though 66% mentioned always practicing social distancing from persons coughing or sneezing. Almost 70% of respondents said they were willing to accept a vaccine with 39% saying they would be willing to pay for it if not publicly funded. In univariate analysis increasing age and having been ever married were associated with GK while tertiary education was associated with GA [Odds Ratio; 95% Confidence Interval] 2.66(1.79–3.95). Independent positive predictors of GK were those who were or had ever been married, those who had marked fear of COVID-19, and had modified their habits in the last three months. Those who had non-tertiary education and had the questionnaire administered as paper rather than online version had GK but age was not a predictor.

**Conclusion:**

Knowledge of transmission and preventive measures should be improved in the general population cognizant of cultural norms and Islamic practices. The study highlights the importance of considering belief systems and perception in developing control measures against COVID-19.

## Introduction

Coronavirus disease (COVID-19) is a disease caused by a newly emerging novel coronavirus called Severe Acute Respiratory Syndrome Coronavirus 2 (SARS-CoV2) that appeared in late 2019 disseminating to cause a global pandemic in 2020. It is related to the SARS-CoV and Middle Eastern Respiratory Coronavirus (MERS-CoV) that emerged in the early 2000s in East Asia and the Middle East respectively. These viruses are of zoonotic origin with SARS-CoV2 thought to have originated in bats. They were not previously identified in humans [1].

Initially, most cases at the epicenter of the outbreak in Wuhan, Hubei province, China had contact with live animals and seafood suggesting animal to human transmission. Later on, person to person spread was reported outside the epicenter [1]. On 31^st^ December 2019, the World Health Organization (WHO) was informed of a cluster of cases of pneumonia of unknown cause detected in Wuhan City, Hubei Province of China. The causative virus of the disease was identified by Chinese authorities on 7th January [2] The WHO declared the outbreak a Public Health Emergency of International Concern (PHEIC) on 30 January, and a pandemic on 11th March [2].

The symptoms of COVID-19 appear after 2–14 days following exposure and vary from asymptomatic, mild symptoms to severe respiratory disease [3]. The main symptoms are fever, cough and shortness of breath. COVID-19 has a much lower case-fatality rate (about 2.67%) or <5% among the confirmed cases, compared with Severe Acute Respiratory Syndrome (SARS), and MERS [4] both of which were not reported in Nigeria. Comorbidities among fatal cases include hypertension, diabetes, coronary heart disease, cerebral infarction, and chronic bronchitis [4]. No specific treatment for COVID-19 is currently available. Clinical management includes prompt implementation of recommended infection prevention and control (IPC) measures and supportive management of complications, including advanced organ support where indicated [5]. Prevention of further spread is of high importance and people should practice frequent hand washing, staying home when sick, and covering their mouths when coughing and sneezing [6]. Until a vaccine is developed, community-based interventions such as school closure, avoiding congregations, adopting social distancing, and creating employee plans to work remotely can help slow the spread of COVID-19 [6]. Many countries enacted travel restrictions to prevent further spread.

From31^st^ December 2019 to 8^th^ June 2020, 6.96million cases of COVID-19 have been reported, including 401 970 deaths in 216 countries worldwide [7]. In Africa there were 189 598 cases; the five countries reporting most cases were South Africa (48 285), Egypt (34 079), Nigeria (12 486), Algeria (10 154), and Ghana (9 638) [7]. Nigeria reported its first case on 27^th^ February when an Italian citizen tested positive for the virus after returning from Italy [8]_._ The virus has affected nearly all the states in the country by 8^th^ June 2020.

In northern Nigeria, Kano the most populous state in Nigeria recorded its first positive case on 11^th^ April, and as of 9^th^ June, there have been 1004 cases, 477 recovered with 49 deaths [9]. Lockdown was instituted in the state on 27^th^ April after “unexplained deaths” of 640 people within the span of 2 weeks [10]. Subsequently, handwashing, social distancing, and masking were made mandatory for all necessary activities in public places across the state.

Kano metropolis is one of the largest in West Africa and also a hub for commercial activities with an estimated population of some 13 million [10]. It is also known for its predominantly Muslim population with many Islamic religious rites and practices requiring congregation and close human interactions e.g., prayers and pilgrimage. However, consequent to COVID-19, the Saudi-Arabian government stopped foreign Muslim pilgrims entering the country for the lesser pilgrimage (Umrah) in an attempt to curtail the spread of the disease. Furthermore, Nigeria directed prospective Umrah pilgrims to defer their plans as recommended to contain the coronavirus [11]. The nature of the virus transmission and control strategies has affected daily activities, livelihoods and liberties, as well as halting several religious obligations, traditional and socio-cultural practices among peoples in many parts of the world. In order to successfully control the pandemic, there is a need to fully understand their knowledge, attitude and practice (KAP), and perceptions. Thus, in this study, we sought to explore the knowledge people have regarding COVID-19, modalities of prevention, attitude, risk perception, the practice of social distancing methods and respiratory hygiene in Kano, Nigeria. It also evaluates the predictors of good knowledge of COVID-19 as well as exploring issues of stigmatization and certain beliefs towards the virus.

## Methodology

### Study design and participants

This was an analytic cross-sectional survey with questionnaires administered to the general population including Health Care Workers (HCW) in Kano, Nigeria. Participants were recruited by convenience sampling.

#### Study setting and sites

Kano is an urban metropolis located in the Northern part of Nigeria and the second-largest city in the country. The study was done in 3 major localities in Kano; Kano Municipal Local Government Area (LGA), Ungogo LGA and, Kumbotso LGA.

### Data collection

The questionnaire was administered in three formats; the first was a paper hardcopy self-administered questionnaire that was distributed in the urban area of Kano accounting for 32.8%, the second was by a trained research assistant interviewer administration in a semi-urban locality32.4% and the last was administered as an online form accounting for 34.8% when social distancing was introduced and interviewer-/self- administration could not proceed as planned. The questionnaire was piloted on 25 random people to ensure the validity and practicability of the questions as conducted in similar studies [12]. Corrections were made and certain relevant questions were added later on as the epidemic progressed into a pandemic. The main survey questionnaire administration was conducted between 12^th^ March to 7^th^ April 2020.

The questionnaire has 4-four components: the demographic data of the respondents, the knowledge of COVID-19, i.e. clinical presentation, risk factors, transmission and prevention, the attitude towards COVID-19 in regards to seeking medical attention and cultural and religious aspects e.g., closure of Mosques, the conduct of pilgrimage, funeral rites and weddings to prevent community transmission. The study started even before the first reported case in Kano and spanned to a point where a state lockdown was put into place. Some relevant questions were added as more information about the disease evolved.

### Statistical analysis

Coded data was imputed into MS Excel and scored. Open-ended questions were analyzed separately, scored responses were summed and used in calculating the total scores of knowledge, attitude and practice for each respondent. Cut-off marks were given that demarcate between respondents that had good knowledge, attitude and practice from those that did not. The demographic data was evaluated and summarized using the descriptive presentation. Quantitative data were presented as mean, standard deviation and inter–quartile ranges. Certain questions were analyzed using simple frequency tables. As in Mallhi et all [12] chi-square and student’s t testing was used in identifying associations between variables and outcomes defined as good knowledge (GK), Good attitude (GA) and Good practice (GP) using score cut-offs of ≥ 75%, ≥ 86.5% and ≥ 75% respectively. A logistic regression model was used to determine independent predictors of GK of COVID 19. The model was constructed using a forward stepwise approach using the Likelihood Ratio Test (LRT) for covariate selection. Covariates were imputed when significant at 20% in univariate analysis. The level of significance (α) was set at 0.05. Analyses was done using Stata version 11.0 (Texas, USA).

Ethics approval was obtained from the Ministry of Health, Kano dated 9^th^ March 2020 with a reference number of MOH/Off/797/T.I/1985. The respondents were interviewed and confidentiality was maintained. All potential participants were provided information about the survey and only those who gave written consent were included in the study.

## Results

### Basic characteristics

**Fig 1:**
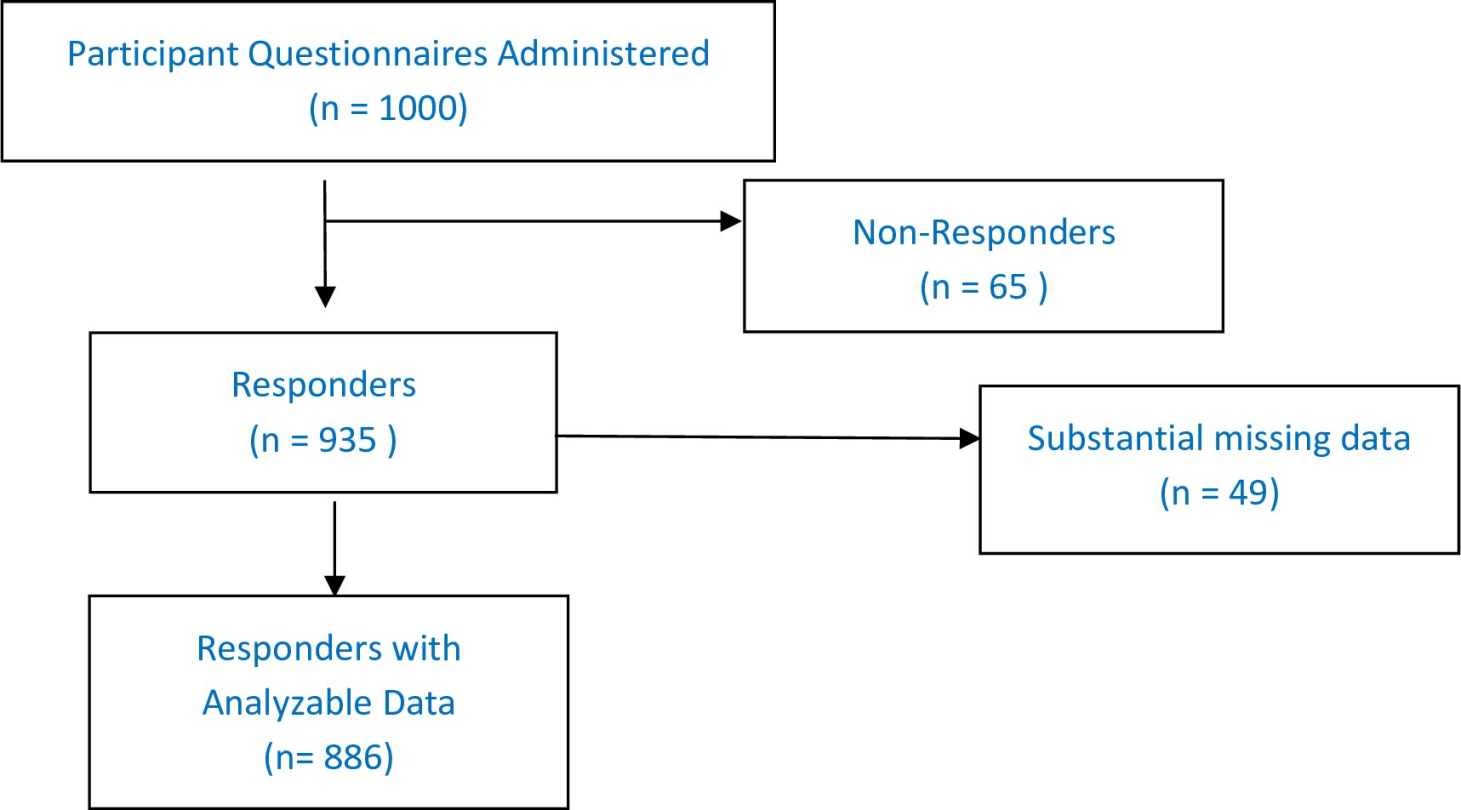
Flow diagram of participants recruitment.

A total of 1000 questionnaires were administered from 12^th^ March to 7^th^ April 2020, and 114 questionnaires were dropped due to non-response or substantial missing data. The total participants who responded were 886 persons with a mean age of 28.58yrs [SD: 10.25], median 26 yrs (IQR: 22yrs– 32yrs) (Fig 1; Table 1). There were males (55.42%), singles (56.32%) while the rest were married or had been married previously. Of the 886, 355 participants had at least a child. Most of the participants were Muslim 93.68%, the others being Christians. Participants had Quranic education 7.2%, Primary school 6.5%, secondary 38.9% and tertiary education 57.34%; out of the latter, 353 respondents were students receiving University education (Table 1). Their occupations were Housewife 87 [9.8%], trader 111 [12.5%], farmer 32 [3.5%], students 353 [39.8%], civil servants 167 [18.9%], health care workers (HCW) 98 [11.1%] and others 32 [3.61%]. The HCWs included doctors 64 [65.3%], nurses 43 [43.9%], lab workers 20 [20.4%], pharmacists 14 [14.3%], hospital attendants 26 [26.5%], physiotherapists 2 [0.02%] and community health workers 30 [30.6%].

**Table 1:** Baseline demographic characteristics of survey participants (n = 886).

SN	Characteristic	Number, n (%)
1	Gender [males/females]	491/395
2	Mean [± SD], years	28.58 (±10.25)
3	Marital Status:	
	Single	499 (56.3%)
	Married	359 (40.5%)
	Divorced	19 (2.1%)
	Widowed	8 (0.9%)
	Separated	1 (0.1%)
4	Religion:	
	Islam	830 (93.5%)
	Christianity	54(6.1%)
	Other	2 (0.2%)
5	Educational Level Attained:	
	Qur'anic	64 (7.2%)
	Primary	58 (6.6%)
	Secondary	256 (28.9%)
	Tertiary	508 (57.3%)
6	Questionnaire Administration:	
	Self-administered	291 (32.8%)
	Interviewer administered	287 (32.4%)
	Online administration	308 (34.8%)

The overall mean [standard deviation] (inter-quartile ranges) for knowledge, attitude and practice scores expressed in percentage were 65.38% [SD 15.90] (IQR: 55.36–77.8%), 71.45% [SD 14.10] (IQR: 60.87–82.61%) and 65.04% [SD 17.02] (IQR: 57.14–76.19%).respectively. Out of the respondents 270 (30.47%) had GK, 158 (17.8%) had GA and 230 (25.96%) had GP using cut-off scores of 75%, 86.5% and 75% respectively. Of the 886 respondents, 648 or (73.10%) identified COVID 19 as a viral disease that affected the lungs and most respondents 686/886 [77.42%] believed breathing infected air was a mode of transmission of COVID 19, while 297/886 (33.52%) said there was a curative treatment; they specified prayer, vaccine and isolation as modes of treatment.

Of the 886 participants, the other modes of transmissions mentioned included–breathing infected air (77.4%), animal-to-human (52.0%), animal to animal (20.4%), human to human (88.6%), environment to human (71.4%), contact with infected surfaces or objects (85%), contact with saliva, nasal secretions, excreta, feces, and fomites of an infected person(75.4%) and contact with live animal markets (34.3%).

The source of information on COVID-19 for most respondents was from social media outlets 437 [49.32%] followed by Radio 381 [43%] and HCWs 215 [24.27%] though some respondents identified more than one source.

Of the 886 respondents, the following were identified as risk groups or activities to disease transmission: HCWs 751 [85%], travel to epidemic areas 792 [89.3%], people who work in live animal markets 411 [46.3%], caregivers 611 [68.9%], visiting clinics where COVID 19 patients are isolated 676 [76.30%] and people of Asian descent irrespective of the region 386 [43.57%]. 348 or [39.2%] participants said certain races were more at risk than others at contracting the disease.

Only about 57% of the respondents thought COVID-19 was preventable and 22.8%% mentioned coughing into one’s hand as a preventive measure.

Of the 886 respondents, the following agreed with these preventive measures: use of face mask (84%), coughing into one’s hands (22.8%), coughing into one’s elbows (67.38%), regular hand washing (89%), use of hand sanitizers (89.62%), travel restrictions to high-risk areas (72.3%) and avoiding touching one’s face with hands (69%).

In regards to attitude to religious norms, 689 [77.77%] agreed on the cancellation of the lesser pilgrimage as a measure to curb the spread of the disease while 209 [23.64%] admitted that greater pilgrimage (Hajj) should proceed despite the persistence of the ongoing pandemic. About 50% of the respondents insisted on attending Friday congregational prayers despite social distancing recommendations and another 38.4% insisted on attending funeral rites. The respondents (60%) attributed the pandemic as a form of God’s wrath and 36% thought it to be a man-made virus. Fifty-four percent (54%) responded that it was a result of increasing contact between humans and animals. As regards to social stigma, about a fourth of the respondents [25.1%] said they would not associate with a person who was discharged after testing negative for the virus while 88% would report a suspected case of COVID 19 to the appropriate authorities. When rating fear on a Likert scale, most respondents [63.5%] had marked fear i.e. ≥ 7 out of 10 and 56% admitted to modifying their habits recently in fear of contracting the virus. Almost 70% of respondents said they were willing to accept a vaccine with 39% of respondents stating they would be willing to pay for it if not publicly funded. In regards to respiratory hygiene and cough etiquette, 51% said they regularly washed their hands and 60% said they avoided congested places. Two-thirds (66.6%) said they used their hand to cover their mouth when coughing though only 20% sometimes used facemask since the onset of the pandemic.

### Basic univariate analysis

Higher proportions of participants with GK were older, married or previously married and responded using a paper questionnaire. Tertiary education was negatively associated with GK while gender had no relationship to it (Table 2), though it was associated with GA [Odds Ratio; 95% Confidence Interval] 2.66 (1.79–3.95).

**Table 2:** Relationship of demographic characteristics to knowledge.

Variable	Good Knowledge n	Poor knowledge	Test-Statistic	P value
Age[years] (mean ± SD	31.98±10.70	27.09±9.68	t = -6.70	<0.0001
Gender [males]	146/270 [54%]	345/616 [56%]	X^2^ = 0.2837	0.594
Marital status [Single%]	88/270 [32.5%]	411/616 [66.67%]	*X*^*2*^ = 88.88	<0.001
Questionnaire type* (paper/paper+online[%])	245/270 [90.74%]	333/616 [54%]	*X*^*2*^ *=* 111.3	<0.001
Proportion of Tertiary education	109/270 [40.37%]	399/616 [64.7%]	X^2^ = 45.69	<0.001

* those who responded using the paper questionnaire compared to online forms

Despite being more knowledgeable about the disease, more participants still agreed on attending wedding ceremonies, burials, Friday prayers and Eid prayers (Table 3). The proportion of respondents who had GK and agreed on cancellation of Hajj if the pandemic persists was 142/223 [63.7%] less than the proportion that had poor knowledge 408/536 [76.1%], (X^2^ = 15.367, <0.0001).

**Table 3:** Relationship of COVID-19 knowledge to social and religio-cultural activities*.

Variable	Good Knowledge	Poor Knowledge	Test Statistic	P value
Agreed on wedding ceremonies [%]	77/161 [47.8%]	55/260 [21.1%]	X ^2^ = 32.86	<0.001
Agreed on attending burial rites despite COVID 19	101/154 [65.6%]	118/265[44.52%]	X^2 =^ 17.31	<0.001
Agreed on attending Friday congregational prayer	131/162[80.8%]	150/267 [56.1%]	X^2^ = 27.19	<0.001
Agreed on attending Eid prayer	112/148 [75.7%]	129/252[51.2%]	X ^2^ = 23.34	<0.001

*Not all respondents participated in this segment

### Multivariate analysis

Independent positive predictors of GK were those who were married or had ever been married, those who had marked fear of COVID-19 and had modified their habits in the last 3 months. Those who had non-tertiary education and had the questionnaire administered as paper rather than online version had GK but age was not a predictor (Table 4).

**Table 4:** Independent predictors of knowledge in multivariable logistic regression.

Predictor of Good Knowledge		Crude Odds Ratio[95% CI]	Adjusted Odds Ratio[95% CI]
Age category [11-19yrs, 20-29yrs, 30-39yrs, 40-49yrs, 50-70yrs]	11-19yrs	1.0 [reference]	1.0 [reference]
	For each increasing age	1.6 [1.40–1.86; p<0.0001]	1.04 [0.84–1.28; p = 0.517]
Marital status	Single	1.0 [reference]	1.0 [reference]
	Ever married	4.15 [3.01–5.71; p<0.0001]	2.56 [1.64–4.03; p<0.001]
Educational level	Non-tertiary	1.0 [reference]	1.0 [reference]
	Tertiary	0.36 [0.27–0.50; p<0.001]	0.81 [0.54–1.20; p = 0.272]
Mode of Questionnaire administration	Paper	1.0 [reference]	1.0 [reference]
	Online	0.12[0.07–0.19; p<0.0001]	0.11[0.07–0.19; p<0.0001]
Fear of COVID 19	Little fear	1.0 [reference]	1.0 [reference]
	Marked fear	0.99 [0.98–0.99; p<0.0001]	2.43 [1.63–3.61; p<0.001]
Modification of habit in the last 3	Modified	1.0 [reference]	1.0 [reference]
	Hasn’t modified	1.00 [0.98–1.01; p>0.05]	0.67 [0.45–0.97; p = 0.04]

Predictors of GK of COVID 19 in Multivariate analysis (Logistic Regression); Log-likelihood ratio = -363.93, R^2^ = 23.77.

## Discussion

This survey conducted in northern Nigeria with predominantly Muslim populations found that less than a third of the participants had GK, GA and GP towards control of COVID-19. It shows advanced or tertiary education and online administration of the questionnaire did not predict GK though current or prior marriage appeared to predict it. Similarly, a significant proportion persisted in their attitudes and practices towards Islamic religious and socio-cultural traditions despite social distancing recommendations. The survey also found one in four persons still harbored stigma towards affected persons even following recovery from the disease.

We found that less than a third of the participants had good KAP scores despite half of them having tertiary education an unexpected ironic finding. The majority knew the causative organism was a virus that primarily affects the lungs and identified breathing infected air as a means of transmission. About half of them were aware of the zoonotic nature of the disease but fewer participants recognized contact with bats as a means of transmission. Only about a third said the disease originated from live animal markets. Half of our respondents said they got their source of information from social media perhaps a reflection of their educational background. About 43% said they also got their information from radio channels while a quarter said they got it from HCWs. This should be noted in planning subsequent community sensitizations and enlightenment.

Similarly, overall knowledge of personal prevention such as hand washing with soap and water, using facemasks and avoiding touching one’s face was good. Unfortunately, appropriate knowledge did not always influence good attitude or practice. However, most respondents [62.2%] agreed with the cancellation of Hajj pilgrimage, one of the fundamentals of Islam, to control the pandemic and prevent further spread of the virus. Nearly a quarter of respondents said they would go ahead, despite social distancing, to attend a wedding ceremony despite over half of them (58%) having GK. We also explored religious obligations and prayers that Muslims attend and found that 38%, 42%, 49% would still go ahead and attend burial rites, Eid prayer and Friday prayer respectively despite existing lockdown measures. These practices predispose the Muslim society to mass community transmission especially since funerals are promptly conducted following a person’s death. However, it appears likely that the risk of transmission is present among those who perform the ritual washing of corpses of infected persons [13].

Additionally, the paper questionnaire respondents were more knowledgeable than online respondents with 90% of people that had GK having had paper questionnaires administered by a trained assistant. This could be due to keyboard psychology, a concept where people online do not feel the need to answer questions correctly due to a degree of anonymity that the computer offers. With the face-to-face interaction of paper-based questionnaire, respondents would be more likely to take time to fill it diligently.

Gender did not have any effect on the degree of knowledge, but increasing age was found to be beneficial in unadjusted analysis. Single individuals were found to have poorer knowledge when compared to respondents who had were married or were ever married. In comparison to non-healthcare workers, HCWs had modified their habits in the preceding three months. Over two-thirds of respondents agreed to accept a vaccine if developed with 80% responding that they would pay for it if not publicly funded.

The current COVID-19 outbreak has provoked social stigma and discriminatory behaviors against people of certain ethnic backgrounds as well as anyone perceived to have been in contact with the virus. Stigma can undermine social cohesion and prompt possible social isolation of groups, which might contribute to further disease spread. This can result in more severe health problems and difficulties in controlling the pandemic [14]_._ More than a third of respondents stated certain races were more at risk of contracting the virus and a fourth harbored lasting stigma. More needs to be done to address such misinformation with correct health information as part of risk communications. It seems that every outbreak comes with its misconception and myths which is further propagated via social media. During the Ebola outbreak in which Nigeria was also affected, at least two lives were lost from drinking salt and water concoctions. This was believed by many to be a form of cure [15]. Across Africa, rumors have been rife that COVID-19 does not affect black people. Another myth was the overzealous intake of vitamin C and Chloroquine would prevent a person from becoming infected which remains unconfirmed [16]. Many conspiracy theories were adduced to COVID-19’s origin particularly in northern Nigeria. Indeed, 36% said that COVID 19 is man-made, 60% said that it was a result of God’s punishment, 18% said that it was an agent of bioterrorism and 54% said increasing contact between humans and animals was responsible. While the proliferation of computers, tablets, and smartphones in the West African sub-region provide channels for the rapid dissemination of information through the internet and social media, the risk of mixing facts with misinformation via these unregulated platforms remains a problem [17].

These findings all support the need for more communication, engagement of communities and local leaders in the promotion of adherence to strict social distancing methods. Religious leaders should be enlightened about the scale of the pandemic and its consequences. Knowledge of transmission of the virus should be communicated clearly and several misconceptions should be clarified and rumors promptly dispelled. Having COVID 19 survivors as ‘champions’ and ‘exemplars’ who talk about their experience will add to the strength of the argument that COVID 19 is real and affecting real people especially in conservative societies such as northern Nigeria.

This study does have limitations given the pace of changing facts and information about COVID 19. Secondly, lockdown measures were instituted middle of data collection, hence the creation of the online format of the questionnaire which may have changed the respondents’ approach to answering questions. Furthermore interviewer effect was not adequately controlled for in the analysis although the logistic regression would have controlled for confounding between imputed covariates. Thirdly, though the study provides essential insight into gaps in knowledge, attitude and practice, the findings, however, should not be generalized as the study was conducted in a sub-Saharan African setting. However, the sample studied is highly likely representative of the Nigerian population.

Since the beginning of our work, several studies of variable sizes have been published on the knowledge, attitude and practice, and perceptions relating to COVID-19. Many of the studies were cross-sectional surveys and were conducted in various populations such as the general public, health care workers, pharmacists, dentists and undergraduate or postgraduate students [18–21]. The modality for questionnaire administration varied from interviewer-administered, self-administered to online self-administration [22] similar to all the three approaches used in our survey. Most studies were conducted in industrialized countries revealing mass media played key roles in higher levels of knowledge, better attitudes and practices [18–20, 23]. Fewer studies have also been conducted in predominantly black African populations e.g., Ethiopia, Rwanda and Uganda [24–26] and also in countries with predominantly Muslim populations e.g., Bangladesh, Egypt and Saudi-Arabia [27–30], with a binational survey conducted in Egypt and Southern Nigeria [28]. As in our study, the results of the surveys generally showed lower levels of knowledge among respondents in developing countries with respondents being less satisfied with their countries’ levels of preparedness towards control of COVID-19 [28]. As in our study, some reports also highlighted the problem of conspiracy theories, discrimination, stigma and xenophobic attitudes especially early in the pandemic and among HIV-infected populations [22, 26, 31]. Our study is the first conducted in a predominantly Muslim area of Nigeria and confirmed lower levels of knowledge. Uniquely the study also highlighted the levels of communal acceptance and likely compliance to intervention measures that restrict Islamic religious rites and practices e.g., Hajj pilgrimage, Muslim congregational prayer, etc. Overall most reviewed studies recommended the need for better and more reliable information and public education [32].

In conclusion Knowledge of COVID-19 is sub-optimal and knowledge of transmission and preventive measures should be improved in the general population cognizant of cultural norms and Islamic practices. The study also highlights the importance of considering belief systems and perception in developing control measures against COVID-19.

## Supporting information

S1 Questionnaire (DOCX)
