# Supplementary material for: Knowledge, attitude and practice survey of COVID-19 pandemic in Northern Nigeria
Source: PLoS One. 2021 Jan 14;16(1):e0245176. doi: 10.1371/journal.pone.0245176 (PMC7808653; doi:10.1371/journal.pone.0245176)
Supplement: S1 Questionnaire — (DOCX) [file pone.0245176.s001.docx]

**KNOWLEDGE, ATTITUDE AND PRACTICE TOWARDS COVID- 19 AMONG THE GENERAL POPULATION IN KANO, NIGERIA.**

SN [_______________]

Name/Initials [_______________________________________] Date [___________________]

A. DEMOGRAPHIC CHARACTERISTICS

1. How old were you on your last birthday? _________

2. Gender (don’t ask) Male [ ] Female [ ]

3. What is your marital status? Single [ ] Married [ ] Divorced [ ] widowed [ ] separated [ ]

4. How many children do you have? _______________

5. Where do you work? ______________________________________________

6. What is your religious faith? Islam [ ] Christianity [ ] Other specify………………..

7. What is your highest educational attainment? Quranic only [ ] Primary [ ] Secondary [ ] Tertiary [ ] Others____

8. What is your main occupation? Housewife; [ ] Trader; [ ] Farmer; [ ] Artisans (Specify______________________) Student; [ ] Civil Servant (not health care worker)[ ]

Health Care Worker [ ] Others (specify) ________________________________________

9. If Health Care Worker specify; Doctor; [ ] Nurse; [ ] Laboratory Worker; [ ] Pharmacist; [ ] Hospital attendant or potter; [ ] Physiotherapist; [ ] Community Health Worker[ ]

SECTION B: KNOWLEDGE OF CLINICAL SIGNS, SYMPTOMS, TRANSMISSION AND TREATMENT

10. Do you know COVID 19 / Coronavirus Disease? Yes [ ] No [ ]; if No go to Question

11. What do you understand by COVID 19 / Coronavirus [____________________________________________________________________]

12. How does COVID 19 / Coronavirus disease manifest?: Fever; [ ] difficulty in breathing; [ ] cough; [ ] runny nose; [ ] sore throat; [ ] Intense fatigue /general weakness [ ]

13. How does COVID 19 spread?

Breathing infected air Yes [ ] No [ ] I don’t know [ ]

Animal-to-human Yes [ ] No [ ] I don’t know [ ]

Animal-to-animal Yes [ ] No [ ] I don’t know [ ]

Human-to-human Yes [ ] No [ ] I don’t know [ ]

Environment-to-human Yes [ ] No [ ] I don’t know [ ]

Contact with infected surfaces or objects Yes [ ] No [ ] I don’t know [ ]

Saliva, nasal secretions, excreta, feces, and fomites of infected person Yes [ ] No [ ] I don’t know [ ]

Contact with Bats Yes [ ] No [ ] I don’t know [ ]

Contact with live animal or seafood Yes [ ] No [ ] I don’t know [ ]

14. Is there Treatment for COVID 19 : Yes[ ]; No [ ]; I don’t know[ ]; if yes specify…………………………………………………………………….

15. What is your source of information on COVID 19? Radio [ ] Health Workers [ ] Friends/Relatives [ ] television [ ] Social media; [ ] Government agency such as ministry of health; [ ] others specify ………………………..

16. Individuals at risk for contracting COVID 19 are:

People who work in live animal markets Yes [ ] No [ ] I don’t know [ ]

Veterinarians Yes [ ] No [ ] I don’t know [ ]

Health care workers Yes [ ] No [ ] I don’t know [ ]

Care givers Yes [ ] No [ ] I don’t know [ ]

Travel to epidemic areas Yes [ ] No[ ] I don’t know [ ]

People of Asian descent irrespective of the region they live in Yes [ ] No [ ] I don’t know [ ]

17. How will the spread of COVID 19 be prevented in the community?

1. Use of face mask Agree[ ] Uncertain [ ] Disagree[ ]
2. Coughing into your hands Agree[ ] Uncertain [ ] Disagree[ ]
3. Coughing into your elbows Agree[ ] Uncertain [ ] Disagree[ ]
4. Regular hand washing Agree[ ] Uncertain [ ] Disagree[ ]
5. Use of hand sanitizers Agree[ ] Uncertain [ ] Disagree[ ]
6. Travel restrictions to high risk areas Agree[ ] Uncertain [ ] Disagree[ ]
7. Avoid touching your face with your hands Agree[ ] Uncertain [ ] Disagree[ ]

Specify…………………………………………..

SECTION C: KNOWLEDGE ON EPIDEMIOLOGICAL RISK FACTORS

18) What are the risk factors of COVID 19?

a) Inhaling infected air: yes [ ]; no [ ]; I don’t know [ ]

b) Direct physical contact with the body of infected persons: yes [ ]; no [ ]; I don’t know [ ]

c) Touching or sharing the linens, clothes, or dishes/utensils of the case: yes [ ]; no [ ]; I don’t know [ ]

d) Sleeping, eating, or spending time in the same household or room as the case: yes [ ]; no [ ]; I don’t know [ ]

e) Travel to places where there is an epidemic: yes [ ]; no [ ]; I don’t know [ ]

f) being a health care worker: yes [ ]; no [ ]; I don’t know [ ]

g) Visiting clinic where COVID 19 patients are isolated: yes [ ]; no [ ]; I don’t know [ ]

h) Direct contact with bats, live animals or sea food: yes [ ]; no [ ]; I don’t know [ ]

19) Are certain people based on race more at risk of getting the disease Yes [ ] No [ ] Uncertain [ ]

SECTION D: PRACTICE

20) How regular do you wash your hands? Always [ ] Often [ ] Sometimes [ ] Rarely [ ] Never [ ]

21) How do you wash your hands? With water [ ] With water and soap [ ] With disinfectant [ ]

22) How often do you wash and disinfect surfaces? Always [ ] Often [ ] Sometimes [ ] Rarely [ ] Never [ ]

23) Do you move away from a congested place when coughing or sneezing? Always [ ] Often [ ] Sometimes [ ] Rarely [ ] Never [ ]

24) Do you distance yourself from a person who is coughing or sneezing ? Always [ ] Often [ ] Sometimes [ ] Rarely [ ] Never [ ]

25) Do you cover your mouth when coughing or sneezing? Always [ ] Often [ ] Sometimes [ ] Rarely [ ] Never [ ]

26) What do you cover your mouth with? hands [ ] inner side of your elbow [ ] tissue[ ] face mask [ ]

27) How often do you use a facemask since onset of epidemic? Always [ ] Often [ ] Sometimes [ ] Rarely [ ] Never [ ]

SECTION E: ATTITUDE

28) Do you think COVID 19 can be prevented Yes [ ] No [ ] Uncertain [ ]

29) Do you think COVID 19 is a serious disease Yes [ ] No [ ] Uncertain [ ]

30) Do you think cancelling the lesser pilgrimage was a good idea to prevent spread of COVID 19 Yes [ ] No [ ] Uncertain [ ]

31) If the epidemic of COVID 19 continues, do you thing Hajj should be cancelled this year ? Yes [ ] No [ ] Uncertain [ ]

32) Would you travel to a high risk region for work/ pleasure Yes [ ] No [ ] Uncertain [ ]

33) What will you do when you have contact with a patient suspected to have COVID 19

a) Take traditional Medicines: yes [ ]; no [ ]

b) Go to Drugs Shop: yes [ ]; no [ ]

c) Go to Hospital: yes [ ]; no [ ]:

d) Do Nothing: yes [ ]; no [ ]

34) What do you advice your families/relatives to do when they are sick?

a) Take traditional Medicines: yes [ ]; no [ ]

b) Go to Drugs Shop: yes [ ]; no [ ]

c) Go to Hospital: yes [ ]; no [ ]

d) Do Nothing: yes [ ]; no [ ]

35) Would you report any suspected case of COVID 19 Yes [ ] No [ ] Uncertain [ ]

36) Would you associate with a person who was infected with COVID 19 but treated and later discharged Yes [ ] No [ ] Uncertain [ ]

37) How would you rate your fear of getting COVID 19 on a 1 to 10 scale with 1 meaning no fear at all and 10 very much fear? 1 2 3 4 5 6 7 8 9 10

No fear at all [ ] Very much fear [ ]

38) How would your colleagues/family members rate their fear of getting COVID 19 on a 1 to 10 scale with 1 meaning no fear at all and 10 meaning very much fear?

1 2 3 4 5 6 7 8 9 10

39) In the past three months have you modified your working habits for fear of getting COVID 19

No [ ]Yes [ ](Specify__________________________________________)

Would you still attend some social gatherings (listed below) despite the COVID 19 pandemic

and local control measures being applied in your area?

1] Wedding Celebration Agree[ ] Uncertain [ ] Disagree[ ]

2] Funeral prayers Agree[ ] Uncertain [ ] Disagree[ ]

3] Friday prayers Agree[ ] Uncertain [ ] Disagree[ ]

4] Eid prayers Agree[ ] Uncertain [ ] Disagree[ ]

Would you accept a vaccine for the COVID 19 if developed? Agree[ ] Uncertain [ ] Disagree[ ]

Please rate your level of acceptance of COVID 19 vaccine

Very unwilling [ ] Unwilling [ ] Not sure [ ] Willing [ ] Very willing [ ]

If the COVID 19 vaccine is not publicly funded, will you be willing to pay for it?

Yes [ ] No [ ]

How much will you be willing to pay for the COVID 19 vaccine?

________________________ naira.

If the cost for the vaccine is higher than what you have just stated what is the maximum amount you are very certain to pay?

________________________ naira.

Do you think COVID 19 is a disease that is ;

1] Man made Agree[ ] Uncertain [ ] Disagree[ ]

2] Due to Climate change Agree[ ] Uncertain [ ] Disagree[ ]

3] Agent of bioterrorism Agree[ ] Uncertain [ ] Disagree[ ]

4] God’s punishment Agree[ ] Uncertain [ ] Disagree[ ]

5] Due to Increasing contact between Humans and Animals Agree[ ] Uncertain [ ] Disagree[ ]

Disagree[ ]
